# The Stepped Care Intervention to Suppress Viral Load in Youth Living With HIV: Protocol for a Randomized Controlled Trial

**DOI:** 10.2196/10791

**Published:** 2019-02-27

**Authors:** Elizabeth Mayfield Arnold, Dallas Swendeman, Danielle Harris, Jasmine Fournier, Leslie Kozina, Susan Abdalian, Mary Jane Rotheram, Adolescent Medicine Trials Network CARES Team

**Affiliations:** 1 Department of Family and Community Medicine UT Southwestern Medical Center Dallas, TX United States; 2 Department of Psychiatry and Biobehavioral Sciences University of California, Los Angeles Los Angeles, CA United States; 3 Section of Adolescent Medicine Department of Pediatrics Tulane University New Orleans, LA United States

**Keywords:** HIV seroposivity, adolescent, young adult, sustained virologic responses

## Abstract

**Background:**

Among youth living with HIV (YLH) aged 12-24 years who have health care in the United States, only 30% to 40% are virally suppressed. YLH must achieve viral suppression in order to reduce the probability of infecting others as well as increasing the length and quality of their own life.

**Objective:**

This randomized controlled trial aimed to evaluate the efficacy of an Enhanced Standard Care condition (n=110) compared to an Enhanced Stepped Care intervention condition (n=110) to increase viral suppression among YLH aged 12-24 years with established infection (not acutely infected).

**Methods:**

YLH (N=220) who are not virally suppressed will be identified at homeless shelters, health clinics, and gay-identified community-based organizations in Los Angeles, CA, and New Orleans, LA. Informed consent will be obtained from all participants. YLH will be randomly assigned to one of two study conditions: Enhanced Standard Care, which includes standard clinical care plus an automated messaging and monitoring intervention (AMMI), or an Enhanced Stepped Care, which includes three levels of intervention (AMMI, Peer Support via social media plus AMMI, or Coaching plus Peer Support and AMMI). The primary outcome is viral suppression of HIV, and YLH will be assessed at 4-month intervals for 24 months. For the Enhanced Stepped Care intervention group, those who do not achieve viral suppression (via blood draw, viral load<200 copies/mL) at any 4-month assessment will “step up” to the next level of intervention. Secondary outcomes will be retention in care, antiretroviral therapy adherence, alcohol use, substance use, sexual behavior, and mental health symptoms.

**Results:**

Recruitment for this study began in June 2017 and is ongoing. We estimate data collection to be completed by the end of 2020.

**Conclusions:**

This is the first known application of an Enhanced Stepped Care intervention model for YLH. By providing the lowest level of intervention needed to achieve viral suppression, this model has the potential to be a cost-effective method of helping YLH achieve viral suppression and improve their quality of life.

**Trial Registration:**

ClinicalTrials.gov NCT03109431; https://clinicaltrials.gov/ct2/show/NCT03109431

**International Registered Report Identifier (IRRID):**

DERR1-10.2196/10791

## Introduction

### Background

New diagnoses of HIV among youth aged 12-24 years continue to be a challenging public health problem in the United States [1,2], with one in four new HIV diagnoses and 60,900 youth living with HIV (YLH) [3]. The substantial improvements in scientists’ ability to prevent and treat HIV infection [4,5] are underutilized by YLH. Similar to adults, if a YLH has an undetectable viral load, there is a 94% likelihood of not transmitting HIV [6] and the YLH is likely to live longer [7] and have a better quality of life [8]. Furthermore, only 30% to 44% of those diagnosed are virally suppressed [2,9]. This study evaluates a Stepped Care model to support YLH to achieve viral suppression.

YLH are concentrated in areas of the United States where the epidemic has grown, with certain groups disproportionately impacted, particularly in the South [10]. Black and Latino men-who-have-sex-with-men are at the highest risk for new HIV infections [11]. Among youth diagnosed with HIV, 81% are gay, bisexual, and transgender youths (GBTY), with the highest rates reported among black and Hispanic/Latino men [2]. GBTY coming of age today may not perceive the same risk of premature death, which characterized young men earlier in the epidemic when there were fewer treatment options.

It is imperative that YLH achieve viral suppression in order to reduce the probability of infecting others as well as increase the length and quality of their lives [7,8]. Among YLH, viral suppression requires linkage and retention in care as well as antiretroviral therapy (ART) adherence. As part of the HIV Treatment Continuum [12], YLH must overcome all barriers to seeking and receiving medical care and then adhere daily to their ART [1,13]. Historically, achieving an undetectable viral load required 95% ART adherence [14]. However, rates as low as 80% may lead to viral suppression [15]; at present, the pills are combined in regimens and ART is more robust. The duration of “drug holidays” (ie, days without medication) is at least as important as the number of pills taken as prescribed when monitoring adherence that aims to result in an undetectable viral load [16]. However, even with only an 80% adherence rate required for viral suppression and the typical regimen consisting of only one pill daily, YLH are far from meeting this target.

Retention in care and adherence to ART are related. Although 41% of YLH know their serostatus, only 62% receive medical care within 12 months of diagnosis [1]. Retention rates can be low, with only one in four YLH retained in care at 3 years after treatment initiation [17]. Among one sample of YLH (atypically, 72% women), initial ART adherence was 69%, but by 1 year, ART adherence was negligible, partly due to only a 30% retention in HIV care [18]. Young people are also more likely to dropout from care than middle-aged and older adults [13,19]. Existing studies suggest that few YLH remain in medical care more than a year, ensuring that ART treatment adherence will also be low [20-22].

### Barriers to Adherence Among Youth Living With HIV

In addition to connecting YLH to care, it is important to identify and address barriers to adherence. Others have suggested that targeting patient group characteristics to improve adherence may not be the answer, but instead, focusing on individual needs with the flexibility to address specific identified barriers may be helpful [23]. Barriers can be structural issues, and many of the challenges to adherence faced by YLH are interrelated. Drawing upon the work of Maslow [24], Barroso and colleagues assert that people living with HIV must first have their basic biological and physiological needs met to become adherent, including food and transportation, and there must be a focus on reducing stigma and addressing community-level barriers that contribute to disparities [25]. Psychological issues can be part of this complex problem, and even forgetting to take one’s medication can be due to the lack of a set routine or a more complex process that involves cognitive or behavioral struggles [26]. Shame as well as mental health problems and substance abuse may also contribute to delayed medication initiation and difficulties with adherence [27]. Thus, it is important that interventions targeting viral suppression have the flexibility to address multiple factors in the lives of YLH.

### Intervention Innovation of the Protocol: Stepped Care

Stepped Care is a strategy used in managing chronic diseases and mental health problems [28-30], but has only recently been applied to YLH in one trial [31]. Using a Stepped Care approach, providers implement the least intensive intervention needed to achieve the treatment goal and intensify the intervention until the treatment goal is achieved. With this Stepped Care framework, this study will implement a low-intensity intervention, followed by sequential introduction of more intense and comprehensive interventions. The interventions at the lowest levels require little individual tailoring and may be sufficient for some youth. However, for YLH who do not achieve viral suppression, a more individualized, tailored intervention will be implemented.

Mobile technologies saturate the lives of youth and young adults and offer an opportunity for a variety of interventions. HIV mobile health interventions are a growing area of interest and have been used as interventions for both self-management and medication adherence [32]. One intervention that may be beneficial for Stepped Care models for YLH is an automated messaging and monitoring intervention (AMMI). AMMI interventions using daily text messaging have been shown to be a useful method for medication reminders for HIV-infected individuals [33] and have demonstrated positive effects on medication adherence [34-40] and viral load [41]. We have demonstrated the efficacy of text messaging interventions with various high-risk groups in prior studies [42-46]. In this study, AMMI is based on promoting ART adherence by enhancing self-management of one’s care using the concepts of social cognitive theory [32,47,48]. Self-management or self-monitoring among persons living with HIV can include reflection, reinforcement of behaviors, and support of cues to action such as taking medications [49]. Peer support is another intervention that is well-suited for use in a Stepped Care model. Positive relationships are a major dimension related to retention in care and adherence to ART medications [50-53]. Relationships provide motivation to increase retention in care for a range of other chronic diseases (eg, diabetes, weight reduction, alcohol treatment, and mental health) [54-56]. Although peer support is a component of many evidence-based interventions, there are mixed findings in the meta-analyses conducted on peer support; some found a significant benefit [57,58] and others found a major benefit [59]. Reviews of peer support studies for persons living with HIV, which aimed at reducing stress [60], have found peer support to be a critical intervention component. In particular, adolescence is a developmental period wherein the influence of peers is crucial and has been consistently recognized as an important period from the 1980s [61] to the most recent Lancet reviews on adolescent health determinants [62].

Coaching interventions adapted for YLH are more intensive, individualized interventions that can be used in a Stepped Care model. The roots of coaching can be traced to sports, where a coach, typically working with a group of youths, is a leader who provides guidance, support, and direction. The earliest mention of coaching in the literature was in the 1970s in relation to assertiveness training [63] and later as a method for improving social skills among children [64] and helping families [65] and other types of groups. Since then, coaching has increased in popularity as an alternative to psychotherapy in the business world [66] and as an intervention for a variety of health professionals. Originally done face-to-face, most recently, this type of intervention, commonly known as “health coaching,” is also delivered electronically [67]. Coaching has been used as an intervention for chronic pain [68], weight loss [69], HIV medication adherence [70], and other health conditions. A recent review of coaching interventions found positive effects in most studies but highlighted the need for research to be more specific in describing coaching interventions including the types of behavior-change strategies used [71].

## Methods

### Overview of Methods and Aims

**Figure 1:**
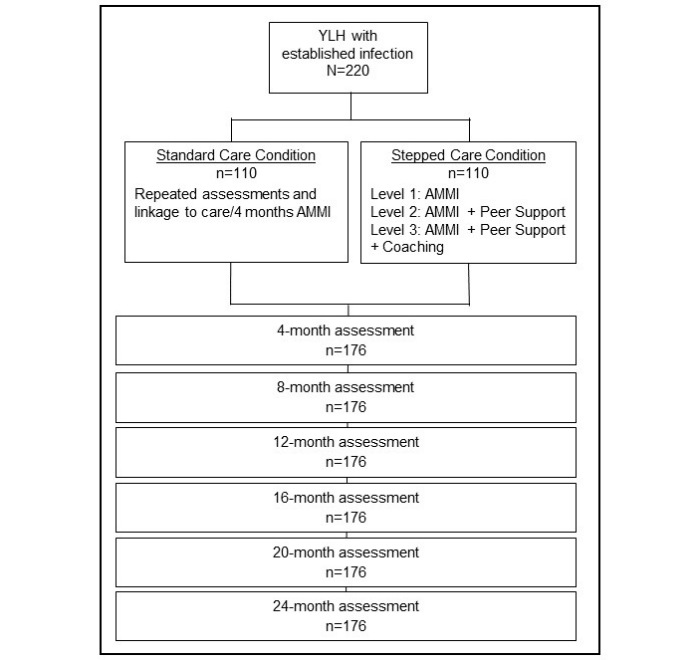
Design of ATN Protocol 148—randomized controlled trial for youth living with HIV (YLH). AMMI: automated messaging and monitoring intervention.

As outlined in Figure 1, we will conduct a randomized control trial (RCT) to evaluate the efficacy of an Enhanced Standard Care Intervention (n=110) as compared to an Enhanced Stepped Care Intervention Model (n=110) in order to significantly increase viral suppression among YLH in two cities, Los Angeles, CA, and New Orleans, LA (Trial Registration: ClinicalTrials.gov NCT03109431). This protocol is part of the U19 Research Program Grant that is funded by the Adolescent Medicine Trials Network for HIV/AIDS Interventions (ATN 148; 1U19HD089886).

The specific aims of this study are (1) to assess if Enhanced Standard Care or an Enhanced Stepped Care results in the primary outcome of more sustained viral suppression and improvements in secondary outcomes, (2) to test whether secondary outcomes such as mental health symptoms and drug use mediate the effect of the intervention on viral suppression over time, (3) and to conduct a cost-effectiveness analysis to weigh the benefit of intervention effects on primary and secondary outcomes against intervention implementation costs beyond costs incurred through the standard of care.

All procedures in this study have been approved by the Institutional Review Board of the University of California, Los Angeles, which serves as the single Institutional Review Board of Record for researchers at the collaborating institutions.

### Recruitment

In both New Orleans and Los Angeles, teams of predominantly bachelor’s degree–level interviewers aim to screen up to 1500 youths to recruit a cohort of 220 seropositive youths. A description of the study sites in Los Angeles and New Orleans can be found in Rotheram-Borus’ and colleagues’ article [12].

### Inclusion and Exclusion Criteria

The current inclusion criteria are age of 12-24 years, HIV-positive status, established HIV infection (not acutely infected), and ability to provide informed consent. Exclusion criteria include age under 12 years or above 24 years, HIV-negative status, acute infection with HIV, inability to understand the study procedures due to intoxication or cognitive difficulties (any youth who appears to be under the influence of alcohol or drugs will be unable to enroll in the study but invited to return at a later date), and inability to provide voluntary written informed consent.

### Recruitment Procedures

To identify eligible participants, youth in high-risk settings will be asked to complete a brief screening and rapid HIV test. All screenings are done face-to-face with a study team member from our Recruitment, Engagement, and Retention Center. Older youths (aged 15-24 years) will provide oral consent for the screening, and younger youths (aged 12-14 years) will provide written consent for the screening to ensure that they understand the screening process. Next, all youths aged 12-24 years whose judgment does not appear to be impaired from a disability or substance use will be asked to give written informed consent to complete the screening. Interviewers are trained to assess for problems with cognition, and additional screening questions will be asked if any concerns are identified. To qualify as “eligible” for recruitment, youths must test seropositive on a rapid HIV test and be virally unsuppressed. Based on these screening criteria, eligible youths will be invited to complete the voluntary informed consent (we have a waiver of parental consent for minors). YHL will be randomized to the intervention conditions via the CommCare system (see next section) after viral load results are obtained and entered, which typically takes several days.

As part of the voluntary informed consent procedures, YHL are asked to provide additional consent to access sensitive information for care coordination and study retention (locating missing participants) for the duration of the study (24 months), which includes their contact information; social media accounts; contact information of their close relatives, friends, and providers including case managers and probation officers; social security number; driver’s license or identification card number; and access to their medical records. Providing this additional information is not mandatory, and YLH can refuse to provide these permissions as long as they provide their own contact information; all information will be stored securely in the CommCare system. Only the study team will have access to this information.

### Assessments

Following enrollment, study participants will be asked to complete a baseline assessment, which is administered by the interviewer using Android tablets in approximately 45 minutes. This assessment covers the following sections: background, risk behaviors, and sociodemographic variables as well as eight cross-cutting domains related to the HIV Treatment Continuum [12]. Interviewers enter the participants’ responses in the CommCare system developed by the Dimagi Corporation, Cambridge, MA. CommCare is an open-source, mobile phone-based platform that is cloud based and HIPAA (Health Insurance Portability and Accountability Act) 1996 compliant. This mobile app is used to collect all study data and send out short message service (SMS) messages and weekly surveys as part of AMMI as well as spontaneous broadcast messages for particular groups of participants.

Participants also have a blood draw for viral load monitoring and a series of rapid diagnostic tests (RDTs) which include the following:

- HIV as part of screening: Potential study participants undergo HIV testing using the CLIA-waived Alere (Waltham, MA) Determine HIV-1/2 Ag/Ab Combo finger stick blood test for both HIV-1/2 antibodies and the HIV-1 p24 antigen with a window period of 12-26 days, which can be read in 20-30 minutes. Once enrolled in the study, participants also receive HIV testing using the Cepheid (Sunnyvale, CA) Xpert HIV-1 Qual Assay, which is highly sensitive, uses whole blood to detect HIV-1 total nucleic acids, and has a 92-minute read time.
- Chlamydia and Gonorrhea are tested using a Food and Drug Administration-approved Cepheid Xpert CT/NG Assay polymerase chain reaction that is read in 90 minutes.
- Syphilis is tested using the CLIA-waived Syphilis Health Check finger stick blood test to detect treponemal antibodies with a 10- to 15-minute read time.
- Hepatitis C is tested using the CLIA-waived OraQuick HCV Rapid Antibody test finger stick blood test with a 20- to 40-minute read time.
- Substance use is tested using a multidrug urine test panel to detect the presence of marijuana, cocaine, opiates, and methamphetamine with a 2- to 5-minute read time.
- Alcohol use is assessed using a breathalyzer test that is sensitive for assessing alcohol use in the last 24 hours.

Following completion of the baseline assessment, participants will receive a US $50 cash incentive. Any study participant who tests positive for HIV or a sexually transmitted infection (STI) will be offered immediate access to treatment.

### Follow-Up Assessments

Study participants will be reassessed every 4 months over a period of 24 months. In total, the study participants will complete six follow-up assessments and receive a US $50 cash incentive for each follow-up assessment. Follow-up assessments will include another 45-minute assessment, a blood draw for viral load monitoring, and a series of RDTs for STIs and substance use, as described above. In addition, if study participants report any potential STI symptoms or exposure, they will immediately be offered testing. In between assessments, YLH who need STI testing can use standard care in the community or contact the study team for assistance. At these assessments, participants who are not virally suppressed (viral load<200 copies/mL) will “step up” to the next level of care while still receiving the prior level(s) of care.

### Outcome Measures

#### Primary Outcome: HIV Viral load

The primary outcome measure is a suppressed viral load at each 4-month assessment for 24 months. At both sites, whole blood is collected with the anticoagulant EDTA and sent at ambient temperature to an internal laboratory where plasma is separated. In addition, 1.5-3.0 mL plasma is shipped to an off-site CLIA-certified laboratory to measure viral load by quantitative HIV-1 RNA real time polymerase chain reaction with a level of detection of 20 copies/mL. Samples from New Orleans are measured at room temperature and those from Los Angeles are measured after they are frozen and thawed. The study team will receive the results through the laboratory’s online portal.

#### Secondary Outcomes

The following secondary outcomes are also measured at each 4-month assessment for 24 months.

##### Retention in Care

YLH are deemed to be retained in care if they attend at least two medical appointments annually. When starting ART, three appointments are scheduled in the first 3 months. Therefore, retention in care also depends on how long YLH have been prescribed ART. We will assess YLH every 4 months; therefore, there are six opportunities over the 24 months of follow-up to obtain self-reports during medical appointments and ensure adherence to ART regimens.

##### Antiretroviral Therapy Adherence

ART adherence is based on a Likert scale that asks YLH to rate their ability to take all their HIV medications as prescribed over the prior 30 days. Response categories range from “Very poor” (1) to “Excellent” (6) [72,73].

##### Alcohol Use Over the Past 4 Months

This parameter will be assessed using the Alcohol Use Disorders Identification Test, consisting of three questions with Likert-scale responses [74].

##### Substance Use

YLH will be asked to indicate if they used any of the following substances over the past 4 months: marijuana, synthetic marijuana, cocaine or crack, heroin, ecstasy, methamphetamines, prescription stimulants or amphetamines, gamma hydroxybutyric acid, ketamine, poppers, inhalants, hallucinogens, prescription painkillers not used as prescribed, and other prescription medications not used as prescribed. YLH will also be asked how many times they injected “drugs such as heroin, opiates, cocaine or amphetamines (crystal)” over the past 4 months. RDTs for alcohol and substance use will be administered to evaluate the degree of underreporting when RDTs indicate use but self-reports do not. Given the fairly short window of detection for RDTs (eg, 24 hours for alcohol), RDTs are not used as outcomes.

##### Sexual Behavior

YLH will be asked to report on the following sexual behaviors over the past 4 months: the number of sexual partners (in total and partners who are HIV positive) and the number of insertive and receptive anal sex acts. YLH will also be asked the frequency of condom use with sexual partners at each sexual encounter. Responses categories range from “None of the time” (0) to “All of the time” (5).

##### Mental Health

Mental health is assessed using three scales. YLH will be administered four questions from the 12-item Short Form Health Survey [75]. Specifically, they will be asked if they “felt calm and peaceful,” had “a lot of energy,” and “felt sad and blue” during the past 4 weeks. Response categories range from “None of the time” (0) to “All of the time” (5). YLH are administered the Patient Health Questionnaire for adolescents (PHQ-A) [76], which asks YLH to report how often they were bothered by problems from a list of nine items over the past 2 weeks. Lastly, YLH are administered the Generalized Anxiety Disorder 7-item scale [77]. Similar to the PHQ-A, YLH are asked to report how often they were bothered by problems from a list of seven items over the past 2 weeks, such as “Feeling nervous, anxious or on edge.”

SMS weekly surveys consist of six items that inquire about how many days YLH felt “sad or depressed;” if they had “any genital itching/pain/discharge, burning during urination, lower stomach pain, or discomfort during sex;” how many times they had sex without using a condom; how many days they used “alcohol and/or drugs;” how many days they did not have a place to sleep; and how many days they missed taking medications. Surveys mainly serve as a tool for research staff to monitor YLH health; check in with them, if needed; and encourage YLH to self-monitor their health and behavior. Items for medication adherence, alcohol/substance use, and sadness/depression will also be treated as outcomes in analyses similar to secondary-outcome analyses. However, low SMS survey–response rates are anticipated based on prior studies with YLH [78]. Therefore, analyses on SMS survey outcomes will be treated as exploratory; precedence will be given to analysis results for secondary outcomes from the 4-month assessment.

##### Costing

Costs are of two types: costs of delivering the intervention and additional costs incurred by participants for their use of health care services and services from other agencies. The cost of delivering the intervention requires an estimation of staff time. Coaching logs and other intervention information will be entered into CommCare, which does not provide information regarding the amount of time spent performing study tasks. Thus, staff activity information that is captured by this system will be supplemented by *Time It*, a mobile app that allows individuals to record the time spent carrying out different staff activities [79]. Each staff member, including program directors, interviewers, and coaches, will record relevant categories of activity over the course of 1 week per quarter. A set of staff activity categories will be prespecified in the mobile app. However, staff are allowed to add more categories as they perform other tasks not previously specified. In addition, personnel time will be estimated from several sources; budgeted time and time recorded on time sheets, for hourly employees, will provide a basis for each staff member’s hours devoted to the project.

The costs of additional services are derived from respondent reports of utilization and medical records and will be estimated using publicly available data. Research-specific costs (eg, incentive payments, informed consent, screens, and software adaptation for survey tools) are excluded from the total costs. All cost data will be adjusted for price back to Year 1 of the study by using the medical care component of the consumer price index.

### Description of the Intervention

As shown in Figure 1, the Enhanced Stepped Care condition has three levels of intervention: Level 1, Enhanced Standard Care plus AMMI; Level 2, online peer support via social media plus AMMI plus Enhanced Standard Care; and Level 3, coaching plus online peer support via social media and AMMI plus Enhanced Standard Care. If YLH fail to be virally suppressed (based on analyses of viral load in the blood stream at 4-month intervals), YLH in the Enhanced Stepped Care condition will be provided the next level of intervention in addition to the other levels of care to which they were previously assigned. Note that these interventions overlap with our team’s other study of HIV-negative youths and are also described in Swendeman et al [49]. To assess dose of the interventions, we will examine the number of responses to the AMMI surveys (Level 1), the number of peer support posts and logins (Level 2), and the number of coaching sessions (Level 3).

#### Level 1

Level 1 of the Enhanced Stepped Care model is Enhanced Care plus AMMI. As this is not a medication treatment study, YLH are treated in the community using best practices that are consistent with available treatment guidelines for providers. However, given that YLH have failed to achieve viral suppression with the existing services available to them, we are enhancing standard care by adding an additional component. Thus, Enhanced Care includes existing services received by YHL from health care providers and other agencies as well as daily text messages. Messages for this study focus on five areas: wellness, health care, medication reminders, drug use, and sexual health. We have tailored and adapted preexisting libraries of theoretically based text messages that have been found to be successful in other RCTs with similar populations [80-83], with messages tailored for two different groups—GBTY and non-GBTY. Messages include a focus on empowering YLH (ie, “Don't rely on other people, take your health seriously,” and “When you take your meds regularly, you’re in control”) as well as providing health-related facts (“Meds keep HIV in check” and “Syphilis can increase your viral load”). The goal of AMMI is to target the areas that can impact adherence to ART that will translate into increased rates of viral suppression.

#### Level 2

The second level of our Enhanced Stepped Care intervention is a secure, private online/social media peer-support intervention. Participants are invited to participate in an online discussion board through muut.com, an open-source discussion forum that is mobile and desktop friendly. Project staff review access requests to ensure that only participants are attempting to join the board. YLH can personalize their Muut profiles using avatars and (nonidentifying) photos, but cannot choose usernames that compromise their anonymity. Online discussion boards are tailored to topics relevant to youth. Others’ experiences with interpersonal group interventions for high-risk youth indicate that group cohesion based on these factors can be a key factor influencing group participation and retention [84,85]. Discussions can be initiated by youth or study team members, including HIV-specific topics related to linkage and retention in care as well as other challenges experienced by YLH.

On all discussion boards, study team members have administrator privileges, allowing them to monitor all activities, post information for discussion, and credit incentives for participants. They take multiple steps to safeguard confidentiality and continually review online postings and delete any identifying information posted. Users are removed from the discussion boards if they post inappropriate content three times after receiving feedback for each occurrence, which includes solicitation for sex and drug use; racist, homophobic, or other stigmatizing content; pornographic content; or “trolling” inflammatory remarks or personal insults.

#### Level 3

Participants who fail to achieve viral suppression at levels 1 or 2 of the intervention will be assigned to our coaching intervention. Coaching will focus on a variety of risk factors concurrently, as is common in HIV research [86-88], and build on our prior work with this type of intervention. Coaching is based on the strength-based model [89,90] that has demonstrated positive impacts on persons living with HIV [91,92] and HIV prevention [93,94]. Identifying and accomplishing goals are critical components of the model. Sessions can be conducted via phone or in person. At the first session, the coach and youth complete a strength assessment that will addresses six main life domains: daily living (survival needs such as food, housing, finances, and employment), physical health (non-HIV-related health problems), health services and health care related to HIV (insurance, linkage to care, ART adherence, viral suppression, treatment cascade, and adherence), social relationships (social support, disclosure, and stigma), mental health (eg, depression, anxiety, and coping), and risks (substance use and risky sexual behaviors). This assessment is used to guide the development of up to three personalized goals with shared responsibility between the youth and coach, depending on the nature of the goals.

After the assessment, sessions will focus on goal attainment. At each session, the coaches use the skills common to 80% of all child and adolescent evidence-based interventions (EBIs) [95,96]: relaxation, relapse prevention, positive activities/alternatives, referrals, modeling/role playing, positive self-talk, triggers, emotional regulation, monitoring/self-monitoring, support networking/building social support, assertive communication, setting up rewards, problem solving, goal setting, praise, and engagement/rapport building. The use of these skills and their relevance to our coaching intervention are described in further detail in Swendeman et al [49]. The coaching intervention is not manualized but rather relies on the strength assessment, goal setting, and the use of evidence-based skills to develop a personalized plan of intervention for each youth that addresses the unique factors that impact adherence and ultimately, viral suppression. Participation in coaching will continue while the YLH is in the study, but we expect that coaching sessions will decrease in frequency over time as the goals are accomplished.

### Selection and Training Coaches

Our coaching model uses paraprofessionals who have experience and skills in working with the population but do not have advanced degrees [97]. These community members have expertise in addressing the “predictable problems” [98] that these youth experience and will use evidence-based practices to intervene and address specific identified goals [97]. Coaches are also hired based on their interpersonal skills, ability to connect with YLH, and skills in implementing an evidence-based but nonmanualized intervention. They receive intensive training prior to field work to participate in ongoing weekly supervision and have access to real-time supervision in the field.

### Data Analyses

#### Aim 1

Intent-to-treat analyses will be used to compare viral suppression as our primary outcome and secondary outcomes between YLH randomized to the Enhanced Stepped Care or Enhanced Standard Care conditions. Comparisons of the same outcomes will also be made between steps in the Enhanced Stepped Care condition as exploratory analyses; the study is only powered for comparisons between study arms. Multilevel models (MLM [99]) will be used to model correlations between repeated observations on the same YLH in order to properly estimate standard errors. Generalized linear MLM will be fit to discrete outcomes such as the binary outcome for viral suppression. MLM fit to the primary outcome for viral suppression will be parameterized to test for the average of viral suppression differences over the follow-up period between study arms (ie, time-averaged effects). This parameterization will be used because all participants are virally unsuppressed at baseline based on eligibility criteria. Secondary outcome levels can differ across study arms; a more standard parameterization that allows for baseline and slope differences over time will be used. We anticipate that randomization will balance out sociodemographic and other important background characteristics across study arms. If characteristics are found to differ across study arms, they will be included as adjustment covariates in MLM. More sophisticated adjustment methods such as propensity scores will be implemented as needed [100]. Data will also be checked for missing data patterns. Appropriate statistical techniques such as multiple imputation will be applied [98,101].

#### Aim 2

We will use bivariate-outcome MLM to examine temporal relationships between primary and secondary outcomes. Model parameterization will be based on a number of factors, including visualizations of outcome trajectories and model fit statistics. One parameterization that may be used in a bivariate-outcome MLM is a bivariate random intercept and slope model that we have used in a prior HIV study to examine the time-varying relationship between HIV-transmission behaviors and mental health symptoms [102]. This model is formulated through two separate MLM equations for each outcome that is linked through random effects to model random intercepts and slopes. A variance-covariance matrix is modeled to estimate correlations between random effects. Correlations capture time-varying associations between outcomes such as the correlation between the first outcome at baseline and the second outcome over time and vice versa. The bivariate-outcome model offers a flexible modeling framework to test different mediational models. For example, if the bivariate-outcome model contains outcomes for viral suppression, a secondary outcome such as mental health symptoms, and an intervention effect covariate, then we can test if mental health symptoms mediate the impact of the intervention on viral suppression. Details on bivariate random intercept and slope models and other model parameterizations to test for mediational effects in longitudinal data are provided in a previous study [103].

#### Aim 3

Cost-effectiveness analyses will be conducted to weigh the benefit of intervention effects on primary and secondary outcomes against intervention implementation costs beyond those incurred through the standard of care. Specifically, analyses will compare the additional cost required, on an average, to obtain an additional unit of outcome in the Enhanced Stepped Care intervention by calculating the cost effectiveness ratio (CER) [104]. The CER is the difference in total costs of providing an Enhanced Stepped Care intervention versus Enhanced Standard Care, divided by the difference in outcomes of Enhanced Stepped and Enhanced Standard Care [104,105]. The primary outcome of viral suppression and secondary outcomes are outcomes of interest. CER is calculated for different combinations of YLH and provider characteristics. We will conduct sensitivity analyses, as recommended by Gold et al [104], to estimate the extent to which the CER calculation is affected by differences in assumptions about the size of the differences in treatment effect. In particular, we will determine how sensitive the CER is to assumptions that the difference in treatment effect is 1 SD below or above the mean estimated effect size. Similarly, we estimate the sensitivity of conclusions to costs that are 1 SD below or above the estimated mean.

#### Sample Size Calculations

Sample size calculations are estimated for our primary analysis, which is a comparison of the probability of viral suppression between Enhanced Stepped and Enhanced Standard Care conditions over five time points. The first follow-up is not included in the comparisons because we anticipate that it will take 6 months for improvements in viral suppression to be seen in the Enhanced Stepped Care condition. Based on our proposed sample size of 220 (n=110 in each condition), we anticipate 80% power for a two-sided test with a .05 alpha level to detect time-averaged percentage differences in viral suppression as small as 9%-13% between conditions. Based on consultation with HIV clinicians, we expect viral suppression rates to be fairly low in the Enhanced Standard Care condition, and we will use a range of rates from 6% to 20% in our calculations. Calculations assume a compound symmetry covariance structure. An autocorrelation coefficient is specified to account for correlations between repeated measurements. Calculations are conducted with a range of autocorrelation coefficient values similar to what we have found in prior studies, from .1 to .3. Lastly, we make a conservative assumption of 20% loss to follow-up so that the sample size in each condition is 88. In practice, we anticipate a much lower attrition rate. Sample size calculations were carried out using Power Analysis and Sample Size software, version 08.0.11 (Englewood, NJ) [106].

## Results

Recruitment for this study began in June 2017 and is ongoing. We estimate data collection to be completed by the end of 2020.

## Discussion

This RCT is the first known application of an Enhanced Stepped Care intervention for adolescents and young adults living with HIV. One of the most innovative aspects of this protocol is one that is slightly more difficult to discern: It will provide guidelines on how to implement evidence-based practices, rather than replicating an EBI manual with full fidelity. Although a great deal of progress has been made in secondary prevention with EBI manuals [107], an Enhanced Stepped Care approach requires assessments to uniquely become part of the intervention process when it is time to step up the intervention. There are individual differences in the need for intervention, and there is no need to provide more intervention than is needed. Instead of each YLH receiving the same intervention (as in many EBIs), the dose and type of intervention are linked to outcomes. Training staff in specific skills allows them to apply these skills based on the needs of the youth in a personalized manner that promotes self-management of one’s health.

Enhanced Stepped Care is a particularly important model since the funding and resources for HIV care have not increased in recent years. If Enhanced Stepped Care is more successful than the Enhanced Standard Care, this model may be a novel way for others to think about their implementation of a typical EBI. It will be critical to establish cost-effective and diffusible strategies that can be nationally diffused; knowing the cost of the two intervention conditions in this study will be important to inform public policy and the selection of interventions by communities. Without a dramatic reversal, HIV incidence among adolescents is expected to increase, and each additional infection costs US $379,668 (in 2010) [108]. The Enhanced Stepped Care model proposed in this study is expected to result in better outcomes and cost savings for society by preventing HIV secondary transmission and postponing disease progression. The clinical benefits of Enhanced Stepped Care without increased costs are documented for diabetes [109] and depression [110]; however, data evaluating the cost-effectives of the Stepped Care model for HIV care are lacking. This study will perform an economic evaluation within the RCT and provide valuable data to support the cost-effectiveness of the Enhanced Stepped Care approach in order to enhance HIV treatment and care among YLH groups from both clinical and societal perspectives. If the Enhanced Stepped Care program results in lower or comparable total health care costs relative to usual care, this finding will offer a unique venture point for scaling up the Stepped Care program across the country.

This protocol is particularly relevant to YLH nationally, who typically face challenges of homelessness, mental health problems, school or job issues, contact with the criminal justice system, and risks within their sexual partnerships in addition to their seropositive HIV status. Studies of ART adherence and retention in care have consistently found depression and the types of life challenges young people are experiencing to be directly related to engagement, retention, and adherence to care over time [16,111]. If we fail to address these comorbid issues with YLH, we will not succeed in meeting the goal of viral suppression with YLH. Our Enhanced Stepped Care approach aims to address these issues with increasingly intensive interventions, based on the individual needs of YLH. Although addressing comorbid issues may be costlier initially, it may have substantial savings in terms of reducing the probability of HIV transmission among YLH, which is an important individual-level and public health outcome.

## Abbreviations

AMMI: automated messaging and monitoring intervention
ART: antiretroviral therapy
CER: cost-effectiveness ratio
EBI: evidence-based intervention
GBTY: gay, bisexual, and transgender youth
HIPAA: Health Insurance Portability and Accountability Act
MLM: multilevel models
PHQ-A: Patient Health Questionnaire for adolescents
RCT: randomized control trial
RDT: rapid diagnostic tests
SMS: short message service
STI: sexually transmitted infection
YLH: youth living with HIV
